# Assessing the filtration efficiency and regulatory status of N95s and nontraditional filtering face-piece respirators available during the COVID-19 pandemic

**DOI:** 10.1186/s12879-021-06008-8

**Published:** 2021-07-29

**Authors:** Deborah Plana, Enze Tian, Avilash K. Cramer, Helen Yang, Mary M. Carmack, Michael S. Sinha, Florence T. Bourgeois, Sherry H. Yu, Peter Masse, Jon Boyer, Minjune Kim, Jinhan Mo, Nicole R. LeBoeuf, Ju Li, Peter K. Sorger

**Affiliations:** 1 Greater Boston Pandemic Fabrication Team (PanFab) c/o Harvard-MIT Center for Regulatory Science, Harvard Medical School, Boston, MA USA; 2 Harvard-MIT Division of Health Sciences & Technology, Cambridge, MA USA; 3 Harvard Ludwig Cancer Research Center and Department of Systems Biology, Harvard Medical School, Boston, MA USA; 4 Beijing Key Laboratory of Indoor Air Quality Evaluation and Control, Department of Building Science, Tsinghua University, Beijing, China; 5 Department of Nuclear Science and Engineering and Department of Materials Science and Engineering, MIT, Cambridge, MA USA; 6 Harvard-MIT Center for Regulatory Science, Harvard Medical School, Boston, MA USA; 7 Computational Health Informatics Program, Boston Children’s Hospital, Boston, MA USA; 8 Department of Dermatology, Yale University School of Medicine, New Haven, CT USA; 9 Environmental Affairs, Brigham & Women’s Hospital, Boston, MA USA; 10 Department of Dermatology, Center for Cutaneous Oncology, Brigham and Women’s Hospital; Dana-Farber Cancer Institute, Boston, MA USA

**Keywords:** N95, KN95, FFR (filtering facepiece respirator), PPE (personal protective equipment), COVID-19, Filtration testing, NIOSH, FDA EUA (Emergency Use Authorization), Occupational health, Regulatory science

## Abstract

**Background:**

The COVID-19 pandemic has severely disrupted supply chains for many types of Personal Protective Equipment (PPE), particularly surgical N95 filtering facepiece respirators (FFRs; “masks”). As a consequence, an Emergency Use Authorization (EUA) from the FDA has allowed use of industrial N95 respirators and importation of N95-type masks manufactured to international standards; these include KN95 masks from China and FFP2 masks from the European Union.

**Methods:**

We conducted a survey of masks in the inventory of major academic medical centers in Boston, MA to determine provenance and manufacturer or supplier. We then assembled a testing apparatus at a university laboratory and performed a modified test of filtration performance using KCl and ambient particulate matter on masks from hospital inventories; an accompanying website shows how to build and use the testing apparatus.

**Results:**

Over 100 different makes and models of traditional and nontraditional filtering facepiece respirators (N95-type masks) were in the inventory of surveyed U.S. teaching hospitals as opposed to 2–5 models under normal circumstances. A substantial number of unfamiliar masks are from unknown manufacturers. Many are not correctly labelled and do not perform to accepted standards and a subset are obviously dangerous; many of these masks are likely to be counterfeit. Due to the absence of publicly available information on mask suppliers and inconsistent labeling of KN95 masks, it is difficult to distinguish between legitimate and counterfeit products.

**Conclusions:**

Many FFRs available for procurement during the COVID-19 pandemic do not provide levels of fit and filtration similar to those of N95 masks and are not acceptable for use in healthcare settings. Based on these results, and in consultation with occupational health officers, we make six recommendations to assist end users in acquiring legitimate products. Institutions should always assess masks from non-traditional supply chains by checking their markings and manufacturer information against data provided by NIOSH and the latest FDA EUA Appendix A. In the absence of verifiable information on the legitimacy of mask source, institutions should consider measuring mask fit and filtration directly. We also make suggestions for regulatory agencies regarding labeling and public disclosure aimed at increasing pandemic resilience.

**Supplementary Information:**

The online version contains supplementary material available at 10.1186/s12879-021-06008-8.

## Background

Filtering facepiece respirators (FFRs) such as N95 masks are the primary mode of respiratory protection for healthcare workers treating infectious agents that are airborne or transmissible via aerosols [1]. As a result of the COVID-19 pandemic, demand for N95 masks and other personal protective equipment (PPE) has greatly outstripped supply, leading to widespread and persistent shortages. In the US, surgical N95 FFRs used in healthcare are regulated by the National Institute for Occupational Safety and Health (NIOSH), a part of the Centers for Disease Control and Prevention (CDC), and by the Food and Drug Administration (FDA; as described in US Code of Federal Regulations 42 CFR part 84 [2]). Similar standards and enforcement mechanisms exist in other industrialized countries [3]. Some FFRs with the filtering properties of healthcare N95 masks, including industrial N95 masks and elastomeric respirators, commonly have exhalation valves. Although ideal for use in other industries, such devices are traditionally not permitted for use in healthcare settings because air exhaled through the valve is unfiltered, precluding the maintenance of a sterile field and providing a possible avenue of disease transmission [4].

High demand for N95 respirators, coupled with disruption of medical supply chains, has led to a severe shortage of respiratory protection for U.S. healthcare workers during the COVID-19 pandemic [5]; news reports describe similar shortages in other countries. In February 2020, the FDA issued the first in a series of Emergency Use Authorizations (EUAs) relaxing regulations on N95 masks to help increase domestic supply [6]. The EUA “NIOSH-Approved Air Purifying Respirators for Use in Health Care Settings,” [7] authorized the use in U.S. healthcare settings of (i) N95 masks manufactured for industrial use and (ii) non-NIOSH approved masks meeting foreign standards functionally equivalent to those for N95 masks. As described in the EUAs “Authorized Imported, Non-NIOSH Disposable Filtering Facepiece Respirators” [8] and “Non-NIOSH Approved Disposable Filtering Facepiece Respirators Manufactured in China,” [9] authorized imported masks include KN95 masks manufactured in China to the GB2626–2006 standard, FFP2 masks manufactured to European standard EN 149:2001 [10, 11], and masks manufactured to trusted performance standards in Australia, Brazil, Japan, Korea, and Mexico among others (we refer to these collectively as N95-type masks). As a practical matter, however, masks from China are the most common.

Manufacturing N95-equivalent masks requires special fabrics and careful quality control. The final products must have three essential functional properties: 1) the ability to filter out small particles (in the case of N95s, 95% of particles of the most penetrating aerosol size tested – canonically 0.3 μm diameter); 2) a tight fit to the face so that inhaled air is directed through the filter fabric and not around the side of the mask; and 3) low inhalation resistance so that a user does not experience difficulty breathing. Unfortunately, data from the CDC [12] and other groups [13] has shown that some respirators manufactured overseas and labeled as N95, FFP2, or KN95 fail to perform as expected for filtration and fit. While this might be a consequence of manufacturing defects, it appears more likely that many of these non-performing respirators are counterfeit or claim adherence to standards that they were not designed to meet [14–16]. In particular, whereas respirators from established Chinese brands have often performed well in quality testing, unfamiliar products and likely counterfeits of known Chinese brands are prevalent in the U.S. supply chain. Unfortunately, fraudulent packaging, poor labelling practices, and insufficient Federal oversight can make it difficult to determine if a given respirator is genuine [17].

The first version of the FDA EUA on “Non-NIOSH Approved Disposable Filtering Facepiece Respirators Manufactured in China” (April 2020) included a list of authorized respirators and vendors in “Appendix A,” but no testing data were required from purported manufacturers to corroborate performance claims. Upon further investigation, many of the companies on this initial list had questionable or unidentifiable websites or business information. The CDC subsequently noted a dramatic increase in counterfeit respirators with labeling that falsely claimed approval by NIOSH or equivalent foreign agencies [14]. On May 7, 2020, in response to a program of performance testing initiated by the CDC demonstrating widespread inadequacies in filtration efficiency, the FDA substantially shortened Appendix A, and on June 6, 2020 the FDA narrowed the scope of authorization to include more specific jurisdictional review requirements [9]. The CDC continues to evaluate masks and to post photographs of the mask packaging for known counterfeit products. On October 15, 2020 the FDA reissued this EUA and updated the list of authorized products on Appendix A. Since that date, the FDA has stopped reviewing new requests for additions of respirator models to Appendix A. This version of Appendix A (issued October 15, 2020) includes 256 FFR models from 167 manufacturers [18].

In this paper, we consider the problem of non-traditional N95-type masks from the perspective of end users involved in healthcare—specifically large teaching hospitals affiliated with Harvard Medical School (HMS). For these users, one consequence of supply chain disruption and the initially permissive FDA EUA is that a large number of unfamiliar models of N95-type masks became available, some through irregular supply chains [19] or donations having unknown provenance. In a healthcare setting, fit testing masks on individual users is standard practice (e.g. using the 3 M FT-30 qualitative fit test kit) [20], but hospitals rarely if ever measure filtration efficiency [19]. Such testing is usually performed by manufacturers, either in-house or by commercial pre-certification laboratories on behalf of manufacturers. In the absence of such capabilities, end users are forced to evaluate masks from dozens of unknown manufacturers based on little or no information. In this paper, we attempt to assess the impact of this lack of information and testing capacity.

We inventoried masks on hand at major academic medical centers in Boston, MA, attempted to match vendors and models to information in Appendix A, and then received a subset of the most common masks (selected by hospital staff) for testing. Performance testing was performed using a simple apparatus—assembled from commonly available components— that can be used to determine if a mask is likely to meet established performance standards. The instrument does not guarantee performance to N95-type standards, but no legitimate mask should fail to exhibit at least 95% filtration efficiency using the apparatus we describe. We also identify multiple labeling and performance problems with non-traditional N95-style masks and formulate a set of recommendations to guide healthcare organizations and other users in assessing mask donations and purchases. Finally, we make suggestions on possible ways the FDA might improve regulatory oversight.

## Methods

**Fig. 1:**
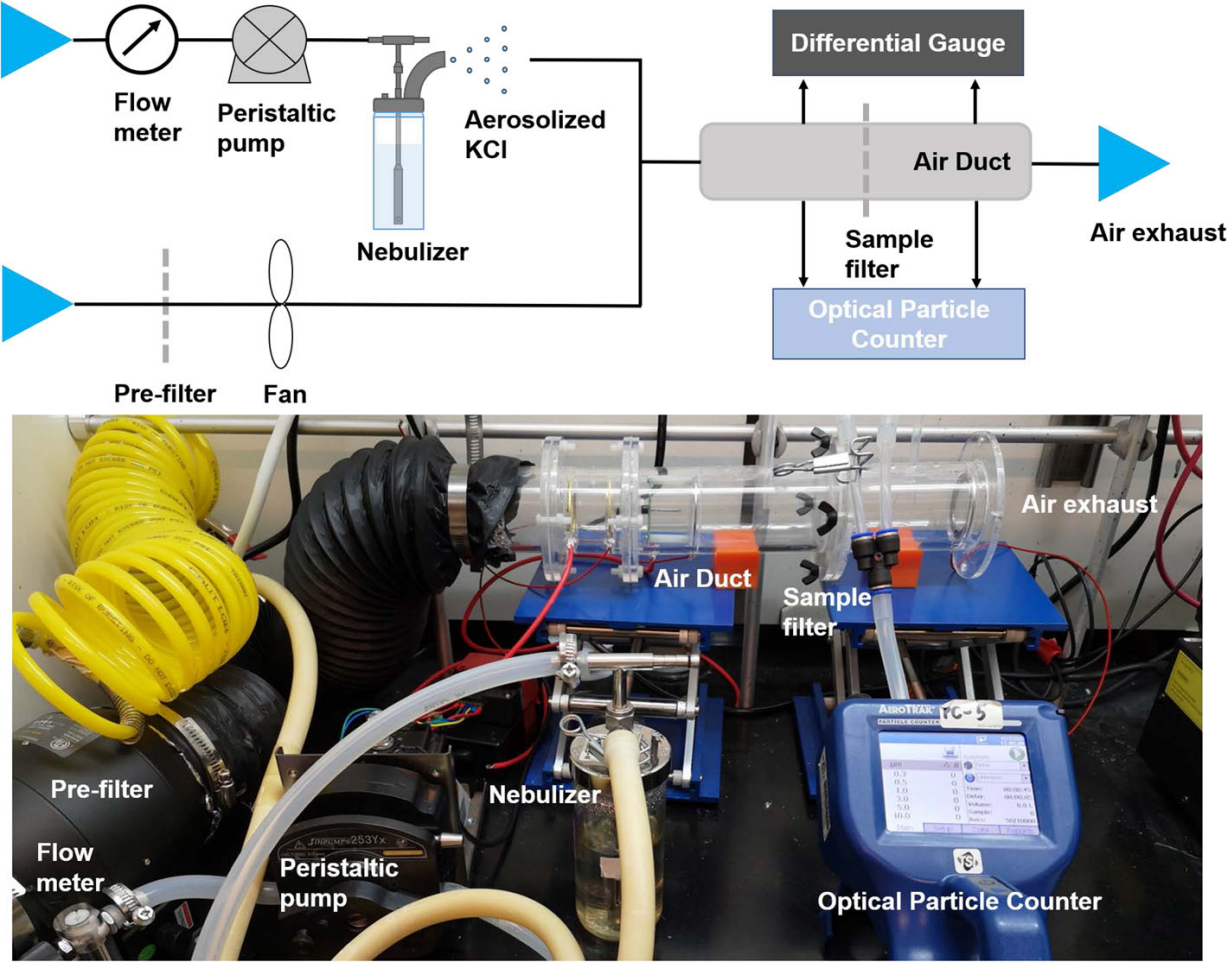
Apparatus assembled from common components and used to test FFRs in this study. Details of the fabrication and use of this device for testing the filtration efficiency of N95-type masks using ambient particles and KCl aerosol particles can be found in the Methods and at http://cleanmask.org/setup. No legitimate FFR should demonstrate less than 95% filtration efficiency using this test, but testing performed with this apparatus is not sufficient to confirm adherence to U.S., European, Chinese or other regulatory standards. Such testing involves greater control over a wider range of test conditions and a formal approach to quality assurance and calibration.

A previously described apparatus for determining the filtration efficiency of N95-type masks was used in this study; this apparatus is designed to replicate NIOSH-approved tests according to TEB-APR-STP-0059 [21] but using equipment available in university laboratories [22, 23]. Square samples 70 mm × 70 mm in size were cut from each mask and inserted into a circular acrylic air duct with an inner diameter of 50 mm (Fig. 1). Either ambient particles or KCl aerosol particles were driven through the respirator filter using air flow to serve as a pollutant source. KCl aerosol was generated by a Collision Nebulizer (BGI Inc., USA) using 10 wt% KCl solution with the volume of free air set at 1 L/min. The concentration of 0.3–10 μm particles was determined using an optical particle counter (Aerotrak 9306, TSI Inc., USA). Concentrations were recorded twice at a one-minute intervals both upstream and downstream of the respirator filter, and the measurements were then repeated once. The single-pass filtration efficiency *η* (*d*_p_) of particles with a size of *d*_*p*_ (μm) was calculated over an 8-min test period as follows:

$$ \eta \left({d}_p\right)=\left(\frac{C_{up}\left({d}_p\right)-{C}_{down}\left({d}_p\right)}{C_{up}\left({d}_p\right)}\right)\times 100\% $$

where *C*_*up*_ and *C*_*down*_ are the average particle counts (pcs) upstream and downstream of the filter, respectively. The pressure drop across the filter was measured by using a differential gauge. The air temperature was nominally *T* = 24 ± 1 °C and relative humidity 30 ± 20%; these values were not controlled but were measured along with air face velocity (which was typically in the range of 0.1 to 0.3 m/s) using a mini thermo-anemometer located at the air duct exhaust. The rate of flow of air through the filter was calculated by multiplying the air face velocity times the cross section of the air duct. For the filters tested under conditions in which face air velocity was < 0.1 m/s (the lower limit of detection for the anemometer), we supplemented data on flow rate with the pressure drop (ΔP), which could be measured more accurately. A single experimenter (E.T.) performed all mask testing described in the study. Further information about the construction and use of this filter-testing instrument, as well as the results of ongoing testing, can be found at http://cleanmask.org.

Filtration efficiency results obtained from the apparatus described above were compared to those obtained to NIOSH specifications at a commercial pre-certification laboratory (ICS Laboratories, USA). Specifically, we tested a set of U.S.-manufactured N95 masks (*n* = 10) that had been exposed to various sterilization procedures as part of a different study [22]. These masks were tested under NIOSH Procedure No. TEB-APR-STP-0059 [21], a method that includes a mask pre-treatment step and utilizes 0.075 +/− 0.02 μm NaCl aerosol particles for filtration performance testing. We observed good concordance between instantaneous filtration efficiency values measured using the two tests, with a Pearson correlation coefficient of 0.89 (*p* = 0.0006) (Additional file1). However, the data described here should be interpreted as a relative, not absolute, measure of filtration efficiency. To further characterize masks being tested we measured their thickness (in mm) using digital micrometer (293–330-30, Mitutoyo America Corp., Aurora, USA) and weight per unit area (grams per square meter; gsm) using a precision balance (AX523/E, Ohaus Corp., Parsippany, USA).

## Results

### Qualitative examination of mask labeling helps identify legitimate respirators

**Fig. 2:**
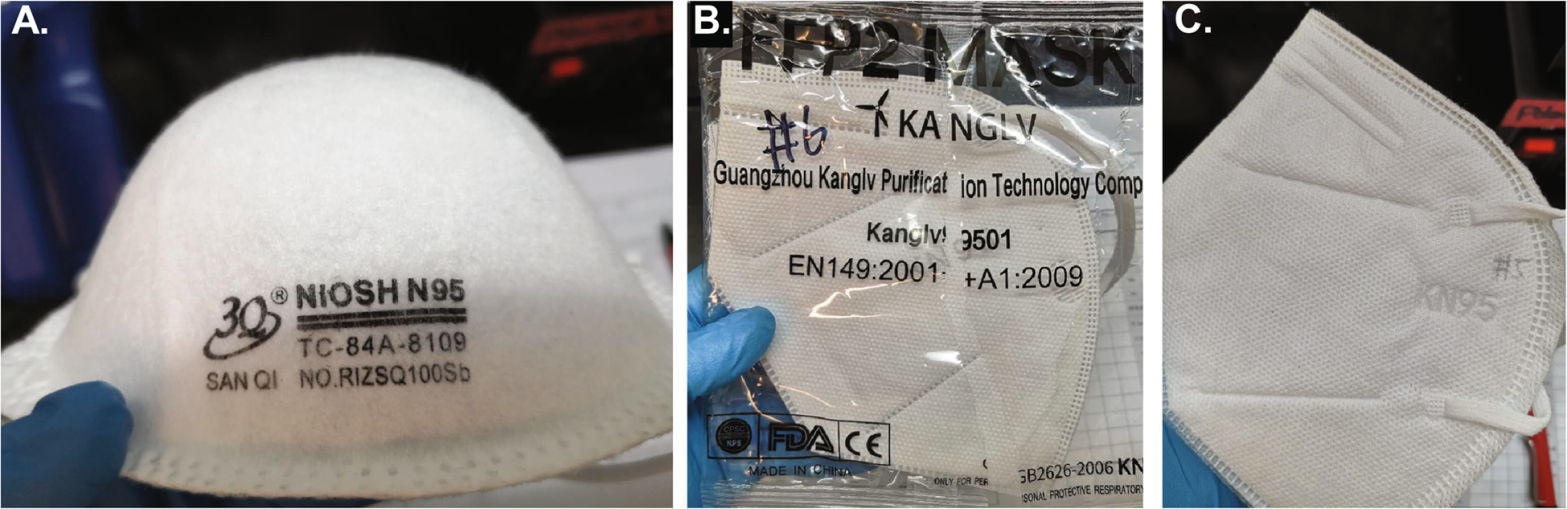
Images of a subset of masks subjected to performance testing and manufactured in China. **a**. A dome-type mask manufactured to N95 standards and listed on the NIOSH website for sale in the US that has all of the required markings. This mask performed as expected (Fig. 3, label # 24). **b**. A flat-fold mask that claims compliance with European FFP2 but contains an FDA logo, which is not allowable. This mask performed well in our tests across all particle sizes and has the performance expected of a legitimate product (Fig. 3, label #2). **c**. A flat-fold mask supplied in bulk with no markings other than the embossed KN95 label; this mask had negative filtration efficiency, and more particles were detected at the output of our test apparatus than at the input (Fig. 3, label #12). Additional mask photographs are available in  Additional file 2.

To assess the diversity of the face mask supply available for use during the COVID-19 pandemic, we inventoried models and makes of N95-style filtering facepiece respirators, many donated, from academic medical centers in Boston, MA. We identified over 100 brands and models in the inventory. In contrast, under standard non-emergency conditions, only two face mask models, both from a traditional domestic manufacturer and provided via a familiar supply chain, would normally be in the inventory of each of the hospitals surveyed (Table 1). A substantial number of the masks on hand originated from unknown vendors and have Chinese writing on the packing, as well as the symbols “KN95” (Fig. 2). Many masks came in packaging that lacked basic information such as manufacturer address, website, and respirator model numbers (Fig. 2c). Visual inspection revealed that some masks in this inventory were similar in appearance or packaging to masks identified as counterfeit by the CDC, as listed on their website [14]. Moreover, a substantial number of masks listed multiple regulatory approvals from different countries. This is problematic because no mask claiming compliance to N95 standards should also claim compliance with KN95 or FFP2 standards; these are different standards, even if functionally similar (e.g. Figure 2b; label #2, Additional file 2). Likewise, CE (Conformité Européenne) markings should only be present on FFP2 masks in conformity with standards for health, safety, and environmental protection of products sold in the European Economic Area [24]. Thus, any mask claiming multiple non-identical regulatory approvals is de facto fraudulent. Nonetheless, the masks we sampled included multiple KN95 masks that were also labelled with CE and NIOSH logos. Several masks included labels such as “PM 2.5,” which typically denotes a lower level of protection from nuisance dust and air pollutants (label #6, Additional file 2). Such masks are likely to have been fraudulently relabeled by stamping “N95” or “KN95” on the box.

**Table 1:** Mask models donated to major academic medical centers in Boston during the COVID-19 pandemic and their corresponding regulatory designation

**N95 - NIOSH Approved**	
Honeywell H901 N95 Particulate Respirator	
3M 1860 N95 Particulate Respirator^c^	
3M 1860S N95 Particulate Respirator^c^	
3M 1870+ N95 Particulate Respirator and Surgical Mask	
3M 8210V Particulate Respirator N95	
3M 8211 Particulate Respirator N95	
3M 8214 N95 Particulate Respirator	
3M 8511 N95 Particulate Respirator	
3M 8511 N95 Particulate Respirator COOL FLOW	
3M 9210/37021 N95 Particulate Respirator^c^	
3M Aura 9211+/37193 Particulate Respirator N95	
3M N95 Drywall Sanding Respirator	
3M Particulate Respirator N95 07048	
3M Particulate Respirator 8000 N95	
3M 9502+ Particulate Respirator N95	
3M Particulate Respirator N95 9211/37022	
Rizhao 3Q SanQi RIZSQ100Sb N95^c^	
AlphaProTech N95 Particulate Respirator Positive Facial Lock	
San Huei SH9550 N95^c^	
Emerald Particulate Respirator N95	
Gerson 1730 N95 Disposable Particulate Respirator^c^	
Gerson 1740 Disposable Particulate Respirator N95	
Gerson 2735 Particulate Respirator N95	
Halyard Fluidshield 3 N95 Particulate Filter Respirator and Surgical Mask	
HDX N95 Respirator Mask Contour Fit	
Jackson R10 Particulate Respirator Dual Valves N95	
Kimberly-Clark N95 62126	
Kimberly-Clark TECNOL 46727 Fluidshield PFR95 N95 Particulate Filter Respirator	
Kimberly-Clark TECNOL 46767 WITH SAFETY SEAL Fluidshield PFR95 N95 Particulate Filter Respirator^c^	
Kimberly-Clark TECNOL 46827 Fluidshield PFR95 N95 Particulate Filter Respirator: Small	
Majestic N95 Particulate Respirator 74-905	
Majestic N95 Particulate Respirator with Valve 74-906	
Milwaukee N95 Valved Respirator	
Moldex 2200 N95 Particulate Respirator	
Moldex 2300 N95	
ONE-fit N95 Healthcare Particulate Respirator and Surgical Mask	
Safety Works N95 Respirator	
SafetyPlus N95 SP8265	
VWR N95 Disposable Respirator	
3M 8200 Respirator N95	
Medicom N95	
3M 8210 & 8210 Plus Particulate Respirator N95^c^	
Medline NIOSH N95	
HDX N95 Respirator Mask	
3M VFLEX N95 Mask	
3M 9010 CN N95 Masks	
Aegis N95 Disposable Respirator	
Aegis N95 Disposable Respirator with Exhalation Valve	
Airtek 972 NIOSH Approved N95	
Cardinal Health N95-ML^c^	
Cardinal Health N95-S^c^	
SAS Safety Corp Particulate Respirator N95	
**KN95 - Appendix A ("Authorized Imported, Non-NIOSH Approved Respirators")**	
3M 9501+ KN95 Particulate Respirator	
aRUN Industrial Co N9 KN95^c^	
Jinan Vhold Co. VH95 KN95^b c^	
Powecom KN95 Protective Mask	
Aoxing KN95^c^	
HuaGuang Communication Disposable Face Mask KN95^b c^	
3M 9541 KN95 Masks	
**KN95 - Not on Appendix A**	
ZKG 9501 KN95	
CM KN-95 White Folding Respirator, 6002A-1 KN95	
FITTOP KN95	
LJK KN95 Stereo Protective Mask	
Urance KN95^c^	
JinLiLaiSi KN-95 Medical Mask	
LanShiOrm KN95 Mask	
Hui Xin KN95 Mask	
DingHang KN95 9001	
BRI-2100 KN95 Mask	
Yuxu (雨旭) 9901 KN95^c^	
Yimengshan (沂蒙山) 9201 KN95^c^	
Energy Fortress KN95 Mask	
SKOOGH KN95	
Graphene SNN70370B KN95^c^	
SNIPPET (施奈邦) SNB9501 KN95^c^	
Dr MFYAN KN95	
Unmarked KN95 (multiple)^c^	
**FFP2- Not on Appendix A**	
3M K112 FFP2^c^	
Guangzhou Kanglv 9501 FFP2^c^	
**Known or Suspected Counterfeit**^a^	
Burvagy Disposable Dust Mask N95 Approved	
Dasheng N95 Respirator Mask DTC3B	
Raxwell N95 Respirator RX9501P	
SANBANG 9051A	
Da Sheng Niosh N95 Respirator Mask DTC3X	
Dasheng N95 Respirator Mask DTC3W	
NIOSH N95 Approved AirTek 9716	
Eco Solutions N95 Particulate Respirator	
**Elastomeric**	
3M 6100/07024 Half Facepiece Reusable Respirators	
3M 6291/07002 P100 Particulate Filters	
**Other Standards (P100, P95, R95, etc)**	
3M 8233 N100 Particulate respirator	
Moldex 4700 N100	
Amston 1802-N99 Particulate Respirator	
3M 8293 P100 Respirator Particulate Mask	
3M 8271 P95 Respirator Particulate Mask	
3M 8247CN R95	
3M 8240 R95	
Cleanwell KF94	
**Not NIOSH Approved or Missing from Certified Equipment List**	
SolidWork Foldable Dust Mask 9600V-N95	
3M 29211 Particulate Respirator	
3M 8102 Mask	
3M Disposable Respirator	
3M Odor Respirator	
3M Performance Filter	
3M Performance Respirator	
AOSafety Sanding Drywall Fiberglass Respirator	
Basecamp Particulate Respirator	
SAS Safety Corp Nuisance Dust Mask	
Shenzhen Health Technology N95	
ESound Med N95 Protective Mask	

N95 model certification was checked in the NIOSH Certified Equipment List

^a^Known counterfeit masks are listed on CDC website; suspected counterfeit masks were identified by guidance listed on the same website

^b^Jinan VHOLD Co LTD VH95 and HuaGuang Communication Disposable face mask were later removed from Appendix A

^c^Mask models that underwent filtration testing at academic medical center

Out of the initial set of over 100 donated brands and models, nineteen of the most commonly donated mask models were selected by hospital environmental affairs staff for further study by our group. Comprehensive testing was not possible due to limitations in personnel and resources during the COVID-19 pandemic. Mitigating this concern is the focus of our work on the properties of products as a class, rather than the performance of specific brands or models.

Masks selected for testing that also had identifiable manufacturer markings included two FFP2, nine KN95, and eight N95 respirators. In addition to the nine KN95 masks with markings, six unmarked KN95 masks that had been provided in bulk were selected for study. N95 masks meeting NIOSH standards must have TC-approval numbers [14] printed on the mask and must be listed on the NIOSH Certified Equipment List (CEL) [25] or the NIOSH Trusted-Source list [26] (e.g. Figure 2a; label #24, Additional file 2). NIOSH has an infographic illustrating the correct labeling of N95 masks that we reproduce in the supplementary materials for convenience (Additional file 3). The NIOSH CEL and Trusted-Source lists pre-date the COVID-19 pandemic and contain information on FFRs that would normally be available through traditional healthcare supply chains; several are manufactured in China and their description conforms to expected standards. Of the eight NIOSH-listed N95 masks we evaluated, four were surgical N95 respirators, which are also subject to clearance by the FDA for healthcare use, and four were industrial N95 respirators, which are not FDA cleared but are currently authorized for emergency use in hospital settings under an active EUA. All eight masks had valid TC numbers and additional information on these models could easily be obtained by searching the manufacturer websites; this may not be a sufficient to demonstrate legitimacy however, because the CDC has identified counterfeit products that borrow TC numbers from legitimate suppliers [14].

Masks claiming compliance with KN95 and FFP2 standards were cross-referenced with the FDA Appendix A list [18] and assessed for a valid business website associated with the brand. Neither of the FFP2 masks in our inventory were listed on Appendix A [18] (Table 1). Data on the 3 M K112 FFP2 mask is readily available [27] and this model appears to be widely distributed in Europe, but we found no reliable information on the Guangzhou Kanglv 9501 FFP2 model (labels #1 and #2 respectively, Additional file 2). Of the nine masks marked KN95 and studied in detail, four were listed on Appendix A initially, but two (HuaGang Disposable face mask, label #6, and Jinan VHOLD Co. VH95, label #7, Additional file 2) were removed as of May 7, 2020, leaving Aoxing KN95 and aRUN Industrial Co. N9 as the only legitimately authorized KN95s in our subset (labels #3 and 4 respectively, Additional file 2); the other five masks could not be matched to any brand or model on Appendix A based on information on the packaging or the mask itself (Table 1; labels # 5 and 8–11,  Additional file 2). Six additional KN95 mask types were completely unmarked and could not be checked against Appendix A or any manufacturer’s website findable by a web search (Fig. 2c; labels #12–17, Additional file 2). Available data on these masks is summarized in Additional file 2 and includes a picture, manufacturer and model (if available), relevant regulatory standard, shape, presence of exhalation valve, type of tethering device, weight, and thickness, in accordance with data types reported in previous studies [28].

### Testing mask performance

**Fig. 3:**
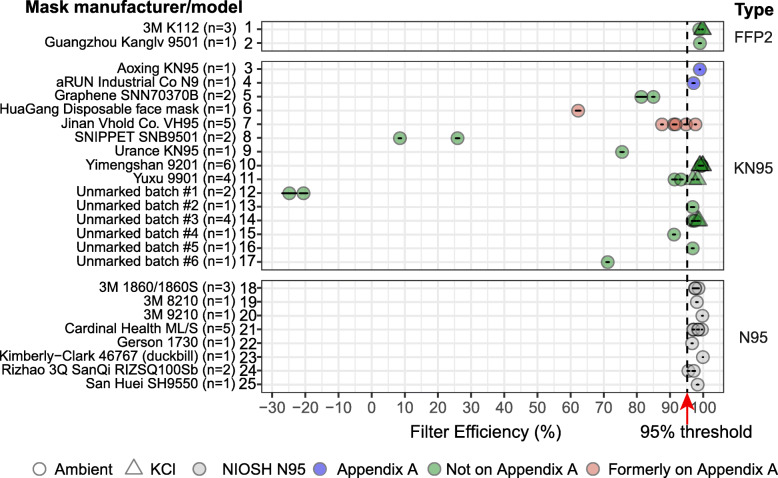
Filtration efficiency of N95-type masks using ambient particles and aerosolized KCl particles as testing agents. Mask filtration efficiency at 0.3 μm particle size is shown. Black lines denote standard deviations of filtration efficiency measurements; full data are provided in Additional file 4. Masks are grouped based on the testing standard they comply with (FFP2, KN95, or N95) but some masks incorrectly claim compliance with multiple standards. “NIOSH N95” refers to masks appearing on the list of “NIOSH-Approved N95 Particulate Filtering Facepiece Respirators” and regulated according to U.S. standards; six of these models are manufactured in the US and the Rizhao and San Huei masks are manufactured in China; all of these masks were available on the US market prior to the COVID-19 pandemic. “Appendix A” refers to masks that are listed in the FDA EUA “Non-NIOSH Approved Disposable Filtering Facepiece Respirators Manufactured in China,” first issued on April 2020 and subsequently updated

We subjected masks to filtration performance testing at a university laboratory as previously described [22, 23] (Fig. 3). Testing was performed on both ambient particulate matter and aerosolized potassium chloride (KCl) in the size range 0.3 to 10 μm, a relevant range for N95 FFRs. Passing this test is not sufficient to establish conformity with NIOSH, EN149, or GB2626 standards, since all three standards involve a range of tests for multiple performance characteristics under carefully controlled conditions [3]. However, results obtained using our testing system conform well to results of tests performed at a commercial pre-certification laboratory to NIOSH standards (see Methods and http://cleanmask.org/procedures for further details). Our testing showed that all N95 masks and a subset of KN95 masks performed as expected in that they removed > 95% of particles down to 0.3 μm from a test airstream. However, a substantial number of KN95 models, both marked and unmarked, failed testing and one unmarked mask released more particles than were present at the input of the testing apparatus, which presents *negative* filtration performance and a potential health hazard (Fig. 3; Additional file 4). The two KN95 masks tested that are still listed on Appendix A exhibited greater than 95% filtration efficiency (Aoxing KN95 and aRUN Industrial Co. N9), whereas the two mask models formerly on Appendix A demonstrated less than 95% filtration efficiency (HuaGang Communication Disposable Face Mask and Jinan VHOLD Co., LTD Model VH95). Additionally, KN95 masks had the largest variability in filtration performance out of all mask types tested, with a filtration efficiency standard deviation of 33.63% as compared to 0.51 and 1.23% for FFP2s and N95s respectively (Table 2). These data are consistent with results from other organizations, including the CDC, [14] showing that poorly performing masks make up a substantial portion of the inventory of non-domestic N95-type masks available in U.S. major academic medical centers. Fortunately, as of this writing, none of the non-performing mask models we studied have been used by a collaborating hospital; they are currently being stored for potential emergency use in the future.

**Table 2:** Average filtration efficiency and standard deviation across mask type. KN95 masks had the largest variability in filtration performance out of all mask types tested, with a filtration efficiency standard deviation of 33.63% as compared to 0.51% and 1.23% for FFP2s and N95s respectively

Mask Type	Mean Filtration Efficiency (%)	Filtration Efficiency Standard Deviation (%)
FFP2 (*n* = 4)	99.3	0.51
KN95 (*n* = 33)	81.1	33.63
N95 (*n* = 15)	98.0	1.23

Fit is a critical feature of N95-equivalent masks and is typically evaluated by end users using OSHA-regulated fit tests to ensure a complete seal with the face [28, 29]. It has been observed that KN95 masks with ear loops instead of headband straps often fail fit testing, and that this feature must be considered when choosing a face mask for healthcare use [14]. We have recently described devices for improving the fit of such masks using secondary mask frames [30]. We also observed that some masks labeled KN95 (a subset of the unmarked KN95s in Fig. 3) have thin perforations and may also have embossed ‘KN95’ lettering that exposes the thin filter layer. This makes the masks particularly fragile and subject to ripping when donned; such masks should be avoided if possible, and carefully inspected before use.

## Discussion

A growing number of investigators and federal agencies have reported that many N95-equivalent masks manufactured overseas, whose distribution in the U.S. became possible due to recent FDA EUAs, do not perform to relevant U.S. and international standards [14]. Our data show that, several months into the COVID-19 pandemic, these under-performing masks made up a substantial portion of the donated inventory at major medical centers in the U.S. (Fig. 3). Our performance testing, although limited in scope, suggests that some masks have inadequate filtration performance for medical use, removing only 8–80% of 0.3 μm particles. Alarmingly, at least one mask added particulate matter to the airstream and therefore had negative efficiency. In many cases these masks purport to be in compliance with multiple non-identical regulatory standards, prima facie evidence that they are counterfeits. Remarkably, some KN95 models that passed preliminary performance testing had little or no identifying markings, or had labelling that was inconsistent with listings in Appendix A. Thus, even what appear to be legitimate KN95 masks can be hard to identify, in part because they lack vendor-specific information similar to the TC numbers required by NIOSH on all N95 masks. We devoted substantial effort to tracking down information on these KN95 masks, but in many cases, we could not find corresponding manufacturers, distributors, or websites. We conclude that it is impossible in many cases to determine whether a KN95 mask is legitimate or not based on the label or packaging. This complicates routine use of KN95 masks in healthcare settings and creates an ideal setting for counterfeiters to produce and misrepresent their products as legitimate. In contrast, we found that all U.S. or Chinese manufactured N95 and FFP2 masks tested in our study exhibited greater than 95% filtration efficiency and adhered to accepted standards for labeling; this typically included an insert or link to a manufacturer’s web site that provided detailed performance data.

### Recommendations for end users

Based on the current study, and in consultation with environmental and occupational health offices at three different hospitals, we propose the following guidelines for sourcing N95-equivalent masks:

1. Use trusted supply chains. Whenever possible, use trusted supply chains to provide products and ask for the technical datasheets or certification documents for a specific brand and model. These documents should not contain obvious spelling or grammatical errors. For all N95 and FFP2 masks that passed our testing, these materials were easily located on manufacturers’ websites.
2. For FFRs claiming N95 certification, check for active and correct TC numbers on the NIOSH Certified Equipment List (CEL) [25] or the NIOSH Trusted-Source site [26]. Check that the TC number matches the style and manufacturer of the mask. Check that all other information matches NIOSH requirements (see infographic in Additional file 3).
3. Check for similarity to a fraudulent product on the NIOSH Web site. We recommend sending pictures of products falsely labelled as “N95” to the CDC so that the agency can expand its online gallery and assist others in identifying products that should not be used under any circumstances [14]. Even seemingly high-quality packaging can hide a nonfunctional product, but low-quality packaging typically denotes a counterfeit product.
4. For FFRs claiming compliance to a non-U.S. standard (e.g. KN95s, FFP2s), check if masks are on the FDA Appendix A [18] or Exhibit 1 [31] lists of respirators authorized for importation under EUA. Also check the CDC Assessment Results for Not NIOSH-approved respirators [12] for filtration performance.
5. Check for inconsistent markings. No FFP2/3, KN95, DS/DL, P2/3, or PFF product should bear a NIOSH stamp since NIOSH only certifies the U.S. N95 standard (the reciprocal is also true). FFP2/3 masks should have a CE mark which indicates conformance to the EU standard, but no KN95, N95, or mask held to a non-EU standard should have a CE mark. A product with multiple labels (KN95, N95, FFP2) is very likely to be fraudulent. A list of different respirator certifications by nation is available at the CDC Website [10].
6. Consider independently performing filtration testing in the absence of verifiable manufacturer information for a specific mask. This can be accomplished by submitting the mask for testing to a CDC or a NIOSH-approved commercial facility (see the CDC International Respirator Assessment Request page [10]). Some institutions may want to consider using their own testing apparatus, as described in the methods section of this paper and at http://cleanmask.org. Fit testing should routinely be performed on all masks used in a healthcare setting.

The possibility that counterfeit masks can have negative filtration efficiency strongly suggests that masks of unknown provenance, or masks whose manufacturer cannot be independently verified, should not be used. For large donations of respirators from unknown suppliers, we recommend that quality assurance testing, including filtration testing, be performed before the respirators are issued to healthcare providers or other frontline personnel. Although we recognize that such testing is difficult to perform for many independent institutions, commercial pre-certification laboratories are able to provide this service at a reasonable cost and turnaround time. For example, the Massachusetts Manufacturing Emergency Response Team (MERT) [32] has collaborated with a network of testing laboratories [33] across the State; their ability to provide functional testing of N95-type masks contributes to community resiliency [34].

## Conclusions

The inconsistent labeling on KN95 masks makes it difficult to identify manufacturers and determine if they are legitimate products. We recommend that all N95-type masks have identifying information printed directly on the product that describes their manufacturer, such as numbers functionally similar to TC numbers for N95 masks (this would require the cooperation of European and Chinese regulators, or an extension of the existing U.S. TC system to all imported products). We also recommend that the FDA make public all data submitted by manufacturers listed in EUA Appendix A. All companies should be required to provide basic operational data including name and place of business, proprietary or brand name, model number, marketing authorization, a copy of the product labeling, and evidence of authorization with quality management systems for healthcare devices (e.g. through 21 CFR Part 820, ISO 13485, or an equivalent) [35]. Any legitimate company will have this information immediately available, although it may initially be provided in a foreign language. Such information is readily available for standard NIOSH-approved N95 masks made in the U.S. and overseas, and this provides a template for Appendix A as well (e.g. a listing of approved surgical N95 manufacturers and models that include links to legitimate corporate websites and donning instructions [36]).

Since the initial issuance of an EUA on N95-style masks, the FDA has thrice amended information on non-NIOSH approved FFRs (in May, June, and October 2020), to improve supply chain oversight. Additional criteria have been established for Appendix A listings to ascertain certification from a trusted notified body. This includes required CE marks for European FFP2 masks and NMPA certification for KN95 masks (National Medical Products Administration, a Chinese government agency for regulating pharmaceuticals, medical devices, and cosmetics [37]). The FDA and CDC have also initiated a large-scale testing program to randomly sample respirators imported from China and test their filtration ability, but this will be of limited use without methods for end users to match information on the foreign-manufactured masks that they have obtained (or intend to purchase) with relevant test results indexed to TC and lot numbers. We also recommend stronger oversight of the respirator supply chain by federal regulatory agencies, including required performance testing of non-NIOSH approved respirators prior to distribution, even in times of crisis. As the current pandemic evolves, generating and maintaining an updated list of trusted alternate suppliers will leave us better prepared for current and future supply shortages.

### Limitations of this study

The testing performed in this study uses equipment available to a university laboratory and is not equivalent to NIOSH-approved testing, in large part because the latter requires highly specialized instruments whose availability remains limited under pandemic conditions. The numerical data in this manuscript should therefore be interpreted as representing relative, not absolute, measures of filtration efficiency. To mitigate this concern, in a related study, we collected data on U.S.-manufactured N95 masks (which had been exposed to various sterilization procedures) using both the testing equipment described in the current work and instrumentation located in a certified commercial laboratory that conforms to NIOSH standards (ICS Laboratories, USA; equipped to perform NIOSH pre-certification testing) [22]. Instantaneous filtration efficiency values measured in the two tests for different masks of the same model undergoing the same sterilization procedure had a correlation coefficient of 0.89 and all masks demonstrating greater than 95% filtration efficiency also passed ICS tests (and vice versa; see http://cleanmask.org). The critical point with respect to the results in this paper is that no functional N95 mask should fail the tests that we perform but a mask passing our tests should not be considered suitable for human use based on our data alone.

As second potential limitation in this study is that the procedures used, including the assembly, calibration and operation of instruments by graduate students and fellows, conform to those used in university-based scientific research but potentially differ from those prevailing in commercial testing laboratories. For example, filtration data were obtained by a single individual who was not blinded to the physical appearance of masks. We did not assign different individuals to mask processing and testing due to limitations on the number of individuals allowed to enter our laboratories; these pandemic-related restrictions remain in place. Mitigating this concern is the fact that key data come from simple, usually digital, instruments and do not involve qualitative interpretation.

A final limitation of this study is that we cannot say whether the specific masks that we tested are representative of the general U.S. market for N95 style masks. Our samples were drawn from a single set of institutions in a single U.S. city using a non-random procedure. However, given that many N95 masks are available on an unregulated grey market it is not clear how objective sampling might be performed except by law enforcement. Moreover, a simple internet search for “N95 mask” continues to yield many products from large retailers (e.g. through Amazon) that have the same misleading labeling as the counterfeit masks analyzed in this study (e.g. claiming KN95 approval by the FDA).

## Supplementary Information

**Additional file 1: ** Analysis of concordance between results obtained from filtration efficiency testing at academic laboratory and ICS laboratories (*n* = 10).

**Additional file 2: ** Attributes of masks undergoing filtration efficiency testing including manufacturer and model, relevant regulatory standard, shape, presence of exhalation valve, type of tethering device, weight, thickness, and a picture of each mask. Mask numbers correspond to label numbers on Fig. 3 and on the first column of Additional file 4.

**Additional file 3: ** NIOSH infographic illustrating the correct labeling of N95 masks.

**Additional file 4: ** Filtration efficiency raw data; depicted in Fig. 3.

## Data Availability

All data generated or analyzed during this study are included in this published article and its supplementary information files.

## Abbreviations

CDC: Centers for Disease Control and Prevention
CEL: Certified Equipment List
EUA: Emergency Use Authorization
FDA: Food and Drug Administration
FFR: Filtering Facepiece Respirator
NIOSH: National Institute for Occupational Safety and Health
PPE: Personal Protective Equipment
