# Supplementary material for: Assessing the filtration efficiency and regulatory status of N95s and nontraditional filtering face-piece respirators available during the COVID-19 pandemic
Source: BMC Infect Dis. 2021 Jul 29;21:712. doi: 10.1186/s12879-021-06008-8 (PMC8319695; doi:10.1186/s12879-021-06008-8)
Supplement: Supplementary file 2 — Additional file 2: Attributes of masks undergoing filtration efficiency testing including manufacturer and model, relevant regulatory standard, shape, presence of exhalation valve, type of tethering device, weight, thickness, and a picture of each mask. Mask numbers correspond to label numbers on Fig. 3 and on the first column of Additional file 4. [file 12879_2021_6008_MOESM2_ESM.docx]

| **Mask Number** | **Picture** | **Manufacturer and model** | **Mask type** | **Shape** | **Approval Standard** | **Regulatory Status** | **Exhalation Valve** | **Tethering Devices** | **Number of masks measured for weight and thickness*** | **Average Thickness (mm)** | **Average Weight (g/m^2^)** |
| --- | --- | --- | --- | --- | --- | --- | --- | --- | --- | --- | --- |
| 1 | 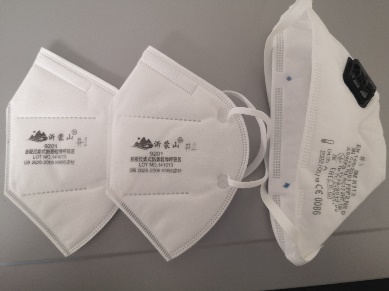 | 3M K112 | FFP2 | duckbill | EN149-2001 | Not on Appendix A | Yes | Headband straps | 1 | 1.05 | 223 |
| 2 | 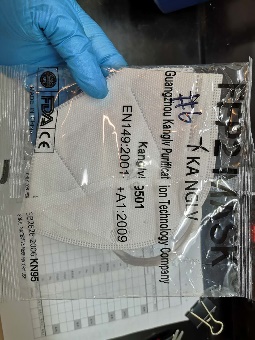 | Guangzhou Kanglv 9501 | FFP2 | flatfold | EN149-2001 | Not on Appendix A | No | Earloop straps | 1 | 0.51 | 111 |
| 3 | 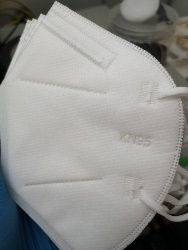 | Aoxing KN95 | KN95 | flatfold | GB2626-2006 | Appendix A  (FDA Authorized) | No | Earloop straps | 1 | 0.85 | 174 |
| 4 | 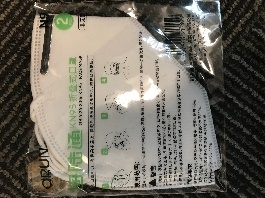 | aRUN Industrial Co N9 | KN95 | flatfold | GB2626-2006 | Appendix A  (FDA Authorized) | No | Earloop straps | 1 | 0.58 | 127 |
| 5 | 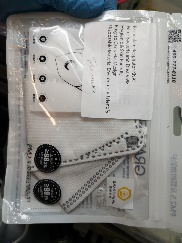 | Graphene SNN70370B | KN95 | flatfold | GB2626-2006 | Not on Appendix A | Yes | Earloop straps | 2 | 0.7 ± 0.03 | 162 ± 4 |
| 6 | 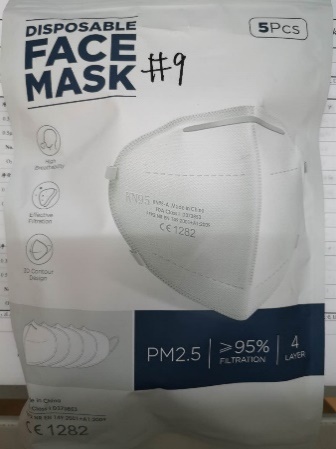 | HuaGang Communication Disposable Face Mask | KN95 | flatfold | GB2626-2006 | Formerly on Appendix A | No | Earloop straps | 1 | 0.67 | 166 |
| 7 | 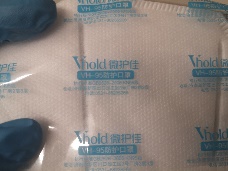 | Jinan Vhold Co. VH95 | KN95 | flatfold | GB2626-2006 | Formerly on Appendix A | No | Earloop straps | 5 | 1.04 ± 0.07 | 233 ± 12 |
| 8 | 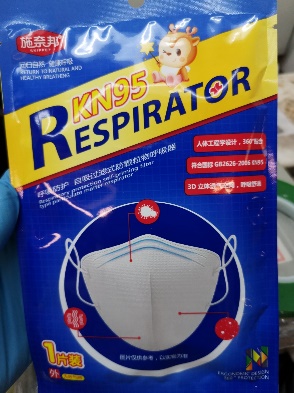 | SNIPPET SNB9501 | KN95 | flatfold | GB2626-2006 | Not on Appendix A | No | Earloop straps | 2 | 0.82 ± 0.03 | 198 ± 6 |
| 9 | 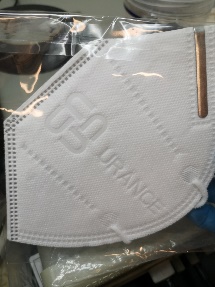 | Urance KN95 | KN95 | flatfold | GB2626-2006 | Not on Appendix A | No | Earloop straps | 1 | 0.56 | 126 |
| 10 | 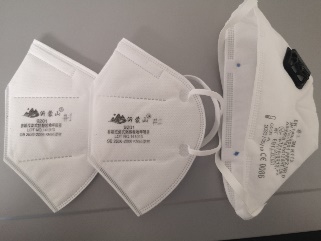 | Yimengshan 9201 | KN95 | flatfold | GB2626-2006 | Not on Appendix A | No | Earloop straps | 2 | 0.83 ± 0.02 | 200 ± 1 |
| 11 | 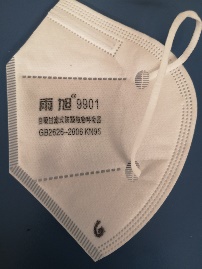 | Yuxu 9901 | KN95 | flatfold | GB2626-2006 | Not on Appendix A | No | Earloop straps | 1 | 0.85 | 189 |
| 12 | 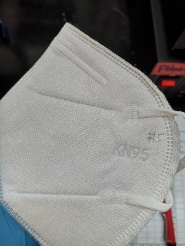 | Unmarked batch #1 | KN95 | flatfold | GB2626-2006 | Not on Appendix A | No | Earloop straps | 1 | 0.75 | 177 |
| 13 | 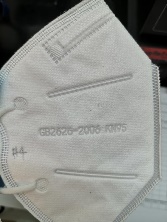 | Unmarked batch #2 | KN95 | flatfold | GB2626-2006 | Not on Appendix A | No | Earloop straps | 1 | 0.74 | 170 |
| 14 | 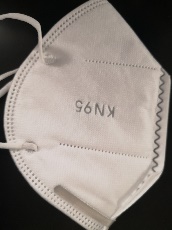 | Unmarked batch #3 | KN95 | flatfold | GB2626-2006 | Not on Appendix A | No | Earloop straps | 2 | 0.81 ± 0.02 | 174 ± 2 |
| 15 | 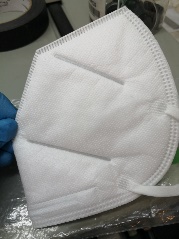 | Unmarked batch #4 | KN95 | flatfold | GB2626-2006 | Not on Appendix A | No | Earloop straps | 1 | 1.04 | 234 |
| 16 | 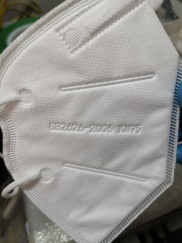 | Unmarked batch #5 | KN95 | flatfold | GB2626-2006 | Not on Appendix A | No | Earloop straps | 1 | 0.73 | 163 |
| 17 | 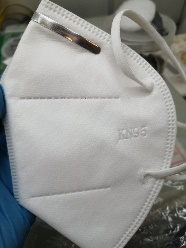 | Unmarked batch #6 | KN95 | flatfold | GB2626-2006 | Not on Appendix A | No | Earloop straps | 1 | 0.72 | 164 |
| 18 | 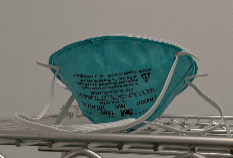 | 3M 1860/1860S | N95 | round | NIOSH-42CFR84 | NIOSH N95 (FDA cleared) | No | Headband straps | 3 | 1.43 ± 0.03 | 316 ± 20 |
| 19 | 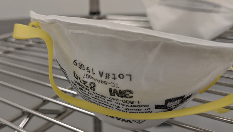 | 3M 8210 | N95 | round | NIOSH-42CFR84 | NIOSH N95 (FDA authorized) | No | Headband straps | 1 | 1.43 | 317 |
| 20 | 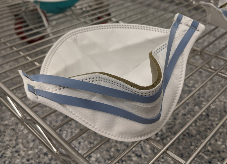 | 3M 9210 | N95 | 3 panel flatfold | NIOSH-42CFR84 | NIOSH N95 (FDA authorized) | No | Headband straps | 1 | 0.85 | 168 |
| 21 | 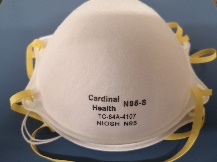 | Cardinal Health ML/S | N95 | round | NIOSH-42CFR84 | NIOSH N95 (FDA cleared) | No | Headband straps | 5 | 1.08 ± 0.12 | 365 ± 28 |
| 22 | 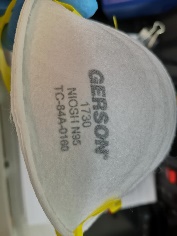 | Gerson 1730 | N95 | round | NIOSH-42CFR84 | NIOSH N95 (FDA cleared) | No | Headband straps | 1 | 1.45 | 328 |
| 23 | 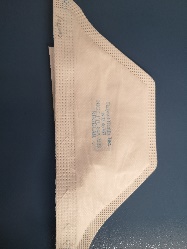 | Kimberly-Clark 46767 (duckbill) | N95 | duckbill | NIOSH-42CFR84 | NIOSH N95 (FDA cleared) | No | Headband straps | 1 | 0.66 | 154 |
| 24 | 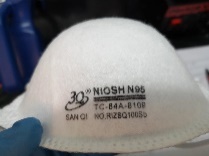 | Rizhao 3Q SanQi RIZSQ100Sb | N95 | round | NIOSH-42CFR84 | NIOSH N95 (FDA authorized) | No | Headband straps | 1 | 1.18 | 306 |
| 25 | 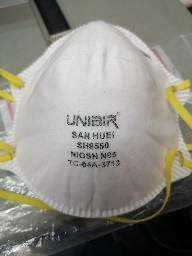 | San Huei SH9550 | N95 | round | NIOSH-42CFR84 | NIOSH N95 (FDA authorized) | No | Headband straps | 1 | 1.02 | 306 |
