# Supplementary material for: Assessing the filtration efficiency and regulatory status of N95s and nontraditional filtering face-piece respirators available during the COVID-19 pandemic
Source: BMC Infect Dis. 2021 Jul 29;21:712. doi: 10.1186/s12879-021-06008-8 (PMC8319695; doi:10.1186/s12879-021-06008-8)
Supplement: Supplementary file 3 — Additional file 3: NIOSH infographic illustrating the correct labeling of N95 masks. [file 12879_2021_6008_MOESM3_ESM.pdf]

Required Labeling of NIOSH-Approved N95 Filtering Facepiece Respirators

For more information about NIOSH-Approved respirators, go to: **http://knowits.NIOSH.gov**

Example of Exterior Markings—Approval holder business name, a registered trademark or an easily understood abbreviation.

If privately labeled, the private label name or logo will appear instead of the approval holder business name.

Model # XXXX—Model Number or Part Number

NIOSH—NIOSH name in block letters or NIOSH logo.

Lot # XXXX—Lot Number and Date of Manufacture (recommended, but not required)

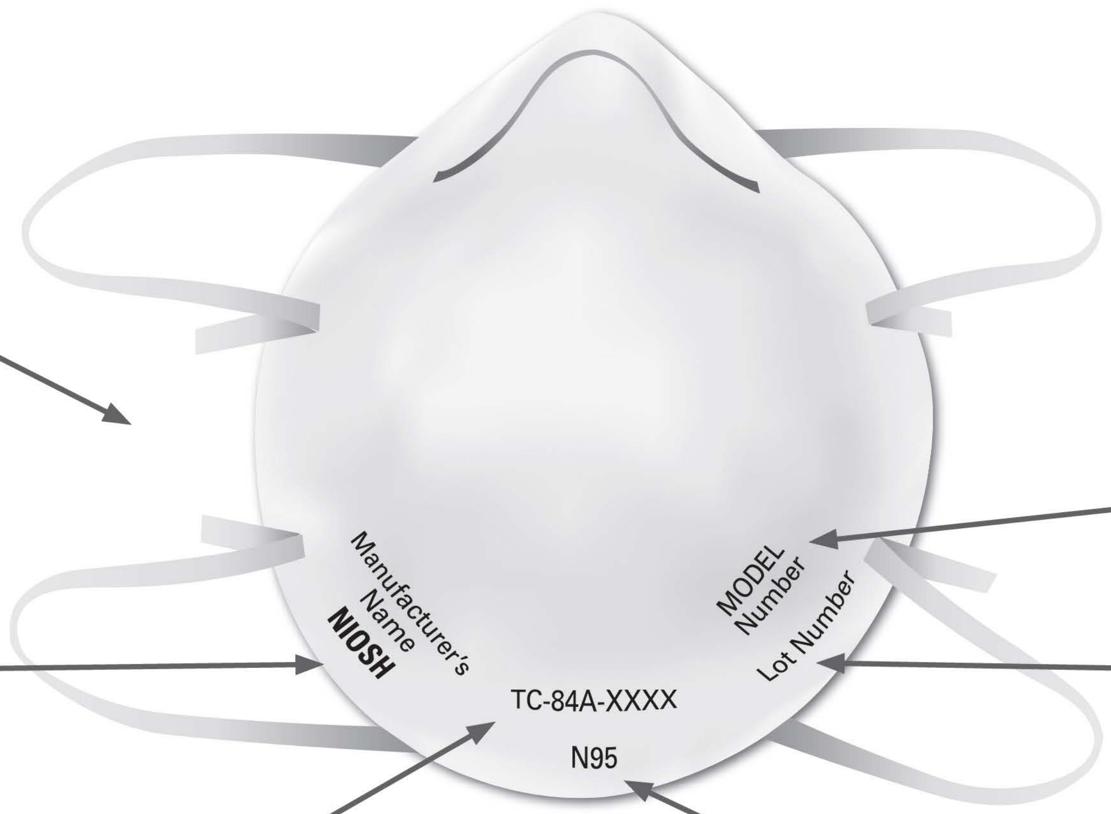

Exterior View

TC-Approval Number (TC-84A-XXXX)—For products manufactured after September 2008, the TC-Approval number is required to appear on the product.

Filter Designation—NIOSH filter series. Alpha-numerical rating followed by filter efficiency level (example, N95, N99, N100, R95, P95, P99, P100)
